# RECOVIR: An application package to automatically identify some single stranded RNA viruses using capsid protein residues that uniquely distinguish among these viruses

**DOI:** 10.1186/1471-2105-8-379

**Published:** 2007-10-10

**Authors:** Dianhui Zhu, George E Fox, Sugoto Chakravarty

**Affiliations:** 1 Dept of Computer Science, University of Houston, 4800 Calhoun Avenue, Houston, TX 77204-5001, USA; 2 Dept of Biology and Biochemistry, University of Houston, 4800 Calhoun Avenue, Houston, TX 77204-5001, USA; 3 Baylor College of Medicine, One Baylor Plaza, Houston, TX-77030, USA

## Abstract

**Background:**

Most single stranded RNA (ssRNA) viruses mutate rapidly to generate large number of strains having highly divergent capsid sequences. Accurate strain recognition in uncharacterized target capsid sequences is essential for epidemiology, diagnostics, and vaccine development. Strain recognition based on similarity scores between target sequences and sequences of homology matched reference strains is often time consuming and ambiguous. This is especially true if only partial target sequences are available or if different ssRNA virus families are jointly analyzed. In such cases, knowledge of residues that uniquely distinguish among known reference strains is critical for rapid and unambiguous strain identification. Conventional sequence comparisons are unable to identify such capsid residues due to high sequence divergence among the ssRNA virus reference strains. Consequently, automated general methods to reliably identify strains using strain distinguishing residues are not currently available.

**Results:**

We present here RECOVIR ("recognize viruses"), a software tool to automatically detect strains of caliciviruses and picornaviruses by comparing their capsid residues with built-in databases of residues that uniquely distinguish among known reference strains of these viruses. The databases were created by constructing partitioned phylogenetic trees of complete capsid sequences of these viruses. Strains were correctly identified for more than 300 complete and partial target sequences by comparing the database residues with the aligned residues of these sequences. It required about 5 seconds of real time to process each sequence. A Java-based user interface coupled with Perl-coded computational modules ensures high portability of the software. RECOVIR currently runs on Windows XP and Linux platforms. The software generalizes a manual method briefly outlined earlier for human caliciviruses.

**Conclusion:**

This study shows implementation of an automated method to identify virus strains using databases of capsid residues. The method is implemented to detect strains of caliciviruses and picornaviruses, two of the most highly divergent ssRNA virus families, and therefore, especially difficult to identify using a uniform method. It is feasible to incorporate the approach into classification schemes of caliciviruses and picornaviruses and to extend the approach to recognize and classify other ssRNA virus families.

## Background

Most non-bacterial epidemic outbreaks are caused by single stranded RNA (ssRNA) viruses. Typically, these viruses undergo rapid genetic mutations that result in a large and dynamic population diversity, which are seen as different virus strains utilizing multiple hosts [1]. Relationships among the strains are usually inferred through conventional homology based comparisons using complete capsid sequences or other genomic regions. Such comparisons seek to identify clusters of similar sequences that comprise major groups (genogroups or genera) and sub-groups (species and serotypes). These groupings are targeted by various diagnostics [2-13] to recognize and classify the viruses.

Four calicivirus genera (noroviruses, sapoviruses, lagoviruses and vesiviruses) and nine picornavirus genera (apthoviruses, cardioviruses, enteroviruses, erboviruses, hepatoviruses, kobuviruses, parechoviruses, rhinoviruses and teschoviruses) are known [14-18]. Further divisions of these genera reflect more detailed sequence relatedness among these viruses. For example, among caliciviruses, noroviruses are divided into two genogroups GI and GII each of which contains seven sequence clusters (GI.1–GI.7 and GII.1–GII.7) [18-21], sapovirus sequences are grouped into 2–5 genogroups, of which, each contains several clusters [22-24], vesivirus sequences are known to contain at least 40 immune response related antigenic serotypes and lagovirus sequences cluster into proposed sero-specific groups [25]. Similarly, the 9 picornavirus genera are classified into several species, each of which consists of a large number of serotypes (Table 1).

**Table 1:** Species and serotypes of all picornavirus genera

**Genera**	**Species #Number of serotypes#**
Aphthoviruses	Foot-and-mouth disease virus (FMDV) #**7**#
	Equine rhinitis A virus (ERAV) #**1**#
Cardioviruses	Encephalomyocarditis virus (EMCV) #**1**#
	Theilovirus (TMEV/TLV/VHEV) #**2 or 3**#
Enteroviruses	Human enterovirus A (HEV-A) #**12**#
	Human enterovirus B (HEV-B) #**36**#
	Human enterovirus C (HEV-C) #**11**#
	Human enterovirus D (HEV-D) #**2**#
	Bovine enterovirus (BEV) #**2**#
	Poliovirus (PV) #3#
	Porcine enterovirus A (PEV-A) #**1**#
	Porcine enterovirus B (PEV-B) #**2**#
	Simian enterovirus (SEV) #**5**#
Kobuviruses	Aichi kobuviruses (AKV) #**1**#
	Bovine kobuviruses (BKV) #**1**#
Parechoviruses	Human parechoviruses (HPeV) #**3**#
	Ljungan viruses #**1**#
Rhinoviruses	Human rhinovirus A (HRV-A) #**75**#
	Human rhinovirus B (HRV-B) #**25**#
Teschoviruses	Porcine Teschoviruses (PTEV) #**11**#

Abbreviations for species are shown within parentheses and the number of serotypes for given species are shown boldfaced within # signs.

The total number of genera, species, serotypes and the associated strains may be taken as a measure of the diversity of a given virus family. Based on this measure, the picornaviruses and the caliciviruses are two of the most highly divergent ssRNA virus families [14,15,17]. Within the genomes of these two virus families, the coat protein sequences show the lowest overall identity, possibly due to the large number of mutations that the viruses undergo to evade host immune responses.

The coat protein consists of a single subunit for the caliciviruses and four subunits (VP1–VP4) for the picornaviruses [15,26]. Structures of picornavirus coat protein [27-31] show that the exposed parts of VP1 subunits contain most of the important neutralization sites [29,32,33] thus making VP1 the immunodominant regions. Additionally, in comparison with the other genomic regions, VP1 sequences show the strongest statistical and phylogenetic support in establishing consistent genetic and antigenic relationships among picornaviruses [15,34]. Therefore, VP1 sequences are most frequently used in molecular evolution and strain characterization studies in picornaviruses.

Although caliciviruses appear to be relatively simpler because of the presence of only one coat protein sequence whose structure is known for a human and an animal strain [35,36], experimental difficulties arise in identifying strains of human caliciviruses as these viruses cannot be antigenically characterized due to their non-cultivability. Consequently, computational techniques offer the most feasible ways to recognize and predict strains of these viruses. Among the different parts of the genome, the coat protein sequences offer the best choice in computation-based strain prediction methods in caliciviruses, mainly because, of their highest overall variability and antigenic correlations as compared with sequences of the other genomic regions. This is true even for the human caliciviruses for which antigenic relationships are often deduced on the basis of antigen and antibody ELISAs using expressed capsid proteins as has been done for noroviruses [21,37-39]. We examine here a computational approach to predict strains in both picornaviruses and caliciviruses on the basis of capsid sequences.

Strain predictions critically depend on sequence information. Most existing prediction methods for caliciviruses and picornaviruses use sequence similarity cut-off values derived from homology-based sequence comparisons between target sequences and known reference sequences [20,34]. Although recent reports indicate reliable estimation of such cut-off values in distinguishing three peak regions that correspond to the genogroups, clusters and strains in noroviruses [40], no uniform criteria exist to accurately estimate these values for the other caliciviruses. Additionally, these cut-off values may be impossible to estimate when different virus genera or families are analyzed together. These difficulties are further compounded when only partial sequences from smaller and relatively more conserved regions are available [12,20,41-44]. Even for complete capsid sequences, homology based similarity scores pose limitations in determining strains of viruses due to the exponential dependence of computation time on the sequence lengths and the number of sequences thereby negatively impacting prediction accuracy.

One approach to address the computational bottleneck is to efficiently align sliding windows of target virus sequences against databases of reference sequences and use the highest overall alignment scores to genotype the target sequences [45]. However, such methods critically depend on parameters such as window sizes and reference sequences. Incorrect choice of these parameters may introduce error-inducing biases while significantly increasing the computation time due to repetitive runs using different trial values of these parameters [45].

Thus, strain recognition methods using sequence identity scores have not been easily amenable to reliable and robust automation across ssRNA virus families. Based on an earlier analysis of noroviruses [46], we describe here an implementation of a residue-wise comparison approach to automate strain predictions using both complete and partial amino acid capsid sequences of caliciviruses and picornaviruses.

## Implementation

### Method

#### Basis

The method is demonstrated using calicivirus and picornavirus capsid sequences. Defining some of these sequences as references, phylogeny based databases of capsid residues that uniquely distinguish among the reference sequences were created. Residue-wise comparisons of the input target sequences with the databases identify those phylogenetic branches whose reference sequences most closely resemble the target sequences. These branches, in turn, yield the genogroup and other classification characteristics of the target sequences thereby identifying their strains.

### Phylogenetic trees of caliciviruses and picornaviruses

Partitioned phylogenetic trees were constructed for each of the 4 calicivirus genera and the 9 picornavirus genera following the procedure described earlier [46] based on evolutionary trace approaches [47]. The virus sequences, obtained from public databases [26,48], were aligned using ClustalW [49] in which the Gonnet-250 distance matrix model [50] was used with penalties for gap opening, closing, extension and separation set to 10, -1, 0.2 and 4 respectively.

The Fitch-Margoliash distance matrix was calculated for each set of aligned sequences and phylogenetic trees were constructed for each such distance matrix using the neighbor-joining Kitsch algorithm of PHYLIP package [51] as implemented on the Cambridge server [52]. The mutation rate was assumed constant throughout the tree. Each tree was displayed with the branches horizontal, the 'root' on the left and the tips on the right.

The topology of these trees, defined in terms of the connectivity and the relative branch lengths, was compared with those of other trees generated using other neighbor joining methods including the unweighted pair group method with arithmetic mean (UPGMA) distance criterion and the minimum evolution criterion using Poisson distance models of amino acid residues as implemented in the MEGA4 package [53,54]. Similar comparisons were also done between the capsid sequence trees and the trees constructed using non-capsid sequences such as those of the RNA-dependent RNA polymerase regions or of the non-coding regions. Because the topology of capsid trees alone remained almost insensitive to the method used, it was deduced that the capsid sequences were phylogenetically most robust. Therefore, any of the neighbor joining methods could be used to draw the trees for capsid sequences, and the Fitch-Margoliash based trees were chosen for further analysis. In contrast, the topologies of the non-capsid trees were quite sensitive to the tree construction method used and hence, these trees were not considered.

### Partitioning of phylogenetic trees yield groups of similar sequences

The trees were divided into ten equally spaced partitions P01–P10. The inter-partition spacing was calculated from the maximum evolutionary distances within the trees. Ten partitions were optimum for all trees as no significant changes in node distributions were observed in any tree with a further increase in partition numbers. The sequences used to construct the trees are called "reference" sequences and the corresponding trees are called "reference" or "genus" trees because each reference tree represents a calici or a picornavirus genus.

**Figure 1:**
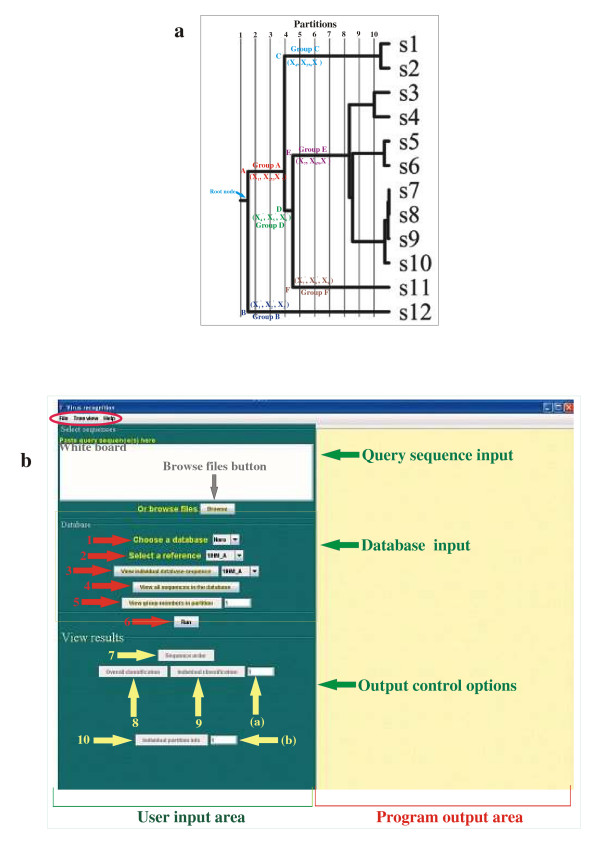
**a: Sequence groups and characteristic residues in partitioned phylogenetic tree**: Representative phylogenetic tree with sequence groups s1–s12 forming the branches of the tree. Each group may consist of one or more sequence clusters. Vertical lines divide the tree into phylogenetic distance based partitions 1–10. Nodes closest to a given partition line and located to the left of the lines define the sequence groups belonging to that partition. Root node and other nodes (A-F) up to partition 5 are shown. Groups corresponding to the different nodes are denoted as "Group n" where n is the node name. For a given partition, characteristic residues (i.e. those residues which are conserved within individual groups but not across the groups) are designated as X and color matched with their node and group names. Subscripts of X denote residue locations which are numbered with respect to a user-defined reference sequence. **b: RECOVIR package GUI screenshot**. Three options are shown in the orange encircled region at the top left hand corner of the GUI; **File**: Allows the output display to be saved in a file; **Tree view**: Displays genus tree in database; **Help**: Provides only limited help as most of the GUI options are self-explanatory through mouse attached tips. The user input and the program output parts of the GUI are indicated at the bottom. The program output part of the GUI displays the output results of the program. The user input area is divided into three parts shown using green arrows: "query sequence input", "database input" and the "output control options". These three parts correspond to the "select sequences", "database" and "view results" options in the GUI. Details of each of these parts are shown using differently colored arrows. The white board/"browse files" button (grey arrow) is used to input query sequence(s). Drop-down menus and toolbars in GUI's database input part are shown using orange arrows along with accompanying numbers; **1: **"Choose a database" drop-down menu allows users to select the input target sequence genus, if known. An "unknown" option may be chosen if the genus is not known; **2: **"Select a reference" allows users to select a reference sequence from the drop-down menu; **3: **"View individual database sequence" option allows users to display a sequence from the selected database; **4: **"View all sequences in the database" option allows users to display all the sequences in the selected database; **5: **"View group members in a partition" allows users to select a partition whose all sequences within the different groups are displayed; **6: **"Run" button allows program activation. The yellow arrows and their accompanying numbers in GUI's "View results" part indicate output display options: **7: **"Sequence order": displays ID number of each input target sequence; **8/9: **Display brief summary/details respectively of partition-wise matches of characteristic residues for an input target sequence identified by its ID in box shown using arrow '(a)'; **10: **Displays details of characteristic residue matches for a chosen partition specified in box shown using arrow (b).

Each partition acts as a "similarity filter" by creating different sequence groups each of which contains similar sequences emanating from a given node within the partition. Examples of such sequence groups are illustrated for a hypothetical representative tree (Fig. 1a). Starting from the root node, partition 1 contains all of the aligned sequences. Sequence comparisons in this partition, therefore, are equivalent to conventional sequence comparisons that consider all of the sequences together. Partitions 2 and 3 are identical and contain the sequence groups s1–s11 and s12 belonging to nodes A and B respectively. Similarly, the two nodes C and D belong to partition 4 while nodes E and F belong to partition 5. Node C of partition 4 contains sequence groups s1 and s2 while node D of the same partition contains nine (s3–s11) of the remaining groups. Similarly, the eight groups (s3–s10) in partition 5 belong to node E while group s11 belongs to node F in the same partition (Fig. 1a). Henceforth, sequence clusters or sequence groups will be referred to simply as groups.

### Comparisons among sequence groups reveal characteristic residues within genus trees

For each tree, sequences belonging to different groups of a given partition were separately aligned and the resulting aligned classes were compared to obtain the consensus residues for that partition. These consensus residues were identified as "characteristic residues" that are conserved within each group but not among the different groups of the partition. For example, characteristic residue X_1 _at location 1 of partition 2 of a given tree may be a conserved Ala for Group A of node A in contrast to a conserved Gly (X_1_') for Group B of node B (Fig. 1a). Such residues were partition-wise generated for each tree.

#### Database creation

Entire information about the genus trees including their partitions, all sequence groups for each partition and all of the characteristic residues taken group-wise, were stored in multiple 2-dimensional arrays that formed the calici and the picornavirus databases.

#### Strain identification through partition-wise comparisons

To identify the strain of the input query ("target") sequence whose genus is known, the program matches the target residues with the characteristic residues of each group of a given partition stored in the appropriate genus database. This is done by first aligning the target sequence with a database reference sequence. Next, starting with the second partition from the root (partition 2 in Fig. 1a), each characteristic residue of a given group in this partition is compared with the target residue at the corresponding location. Such comparisons are carried out for all partition 2 groups. The input target sequence is assigned to the partition 2 group with the maximum number of matches.

The program proceeds to the next partition, where, instead of testing all partition groups, the program tests only those that are directly tree-linked with the most recently accepted group. This greatly reduces the number of groups to be searched. The process continues until all partitions have been searched. Testing only a limited number of connected groups per partition guarantees an optimal tree search time, thereby making the program computationally efficient.

Within a given partition, once input target sequence residues are matched, they are flagged as "marked" and not considered in subsequent partitions. Exceptions to the flagging procedure are carried out only in case of ambiguities. For example, if all of the groups in a partition show an identical number of characteristic residue matches, an ambiguity is declared and no match is flagged. This ensures that all such matched residues of the input sequence are available again for matching purposes in subsequent partitions thereby helping resolve the ambiguity. Ambiguities may also occur when all groups within a given partition show no matches with the input sequence, or, if two successive partitions show identical numbers of characteristic residue matches. In both these cases, the program ignores the ambiguous partition(s) and proceeds to the next one without marking any residue. This allows all of the unmarked residues in the current partition to be compared in subsequent partitions.

To illustrate the method, consider an input sequence corresponding to a known genus whose characteristic residues X_1_, X_2 _and X_3 _for partition 2 are Ala, Pro and Ser respectively for group A and the corresponding group B residues (X_1_', X_2_' and X_3_') are Gly, Thr and Met respectively (Fig. 1a). If the aligned target sequence shows more matches for X_1_, X_2 _and X_3 _of group A in comparison with that of X_1_', X_2_' and X_3_' of group B, it implies that the input sequence belongs to group A in partition 2 and not to group B. Thus, residues X_1_, X_2 _and X_3 _are flagged and comparisons in subsequent partitions follow along those branches that are connected to group A (Fig. 1a). The program therefore proceeds to groups C and D in partition 4 ignoring partition 3 as it is identical to the previous partition 2 (Fig. 1a).

In partition 4, if both groups C and D show equal number of matches i.e. say, X_4 _and X_4_' are both Trp and X_5 _and X_5_' are both Leu (Fig. 1a) and the aligned target sequence also contains Trp and Leu at these locations, then the program will not flag these characteristic residues but will instead carry them over to partition 5 where these residues will again be matched in groups E and F (Fig. 1a) to determine which of these two groups maximally matches the target sequence. Similar comparisons in subsequent partitions 6–10 unambiguously identify the database strain that most closely resembles the input target sequence thereby yielding its strain characteristics.

When the genus of the target sequence is not known, the sequence is first compared with groups of representative reference sequences (≤ 3 sequences per group) from each of the genus trees in the database using ClustalW [49]. Alignment scores are computed for each group. The highest alignment score is used to select the most appropriate genus tree from the databases. Detailed strain identification is then conducted as described earlier. The use of a small number of representative sequences per group ensures rapid genus determination regardless of the number of reference sequences present in the genus tree.

#### Detecting recombination and spontaneous mutations

**Figure 2:**
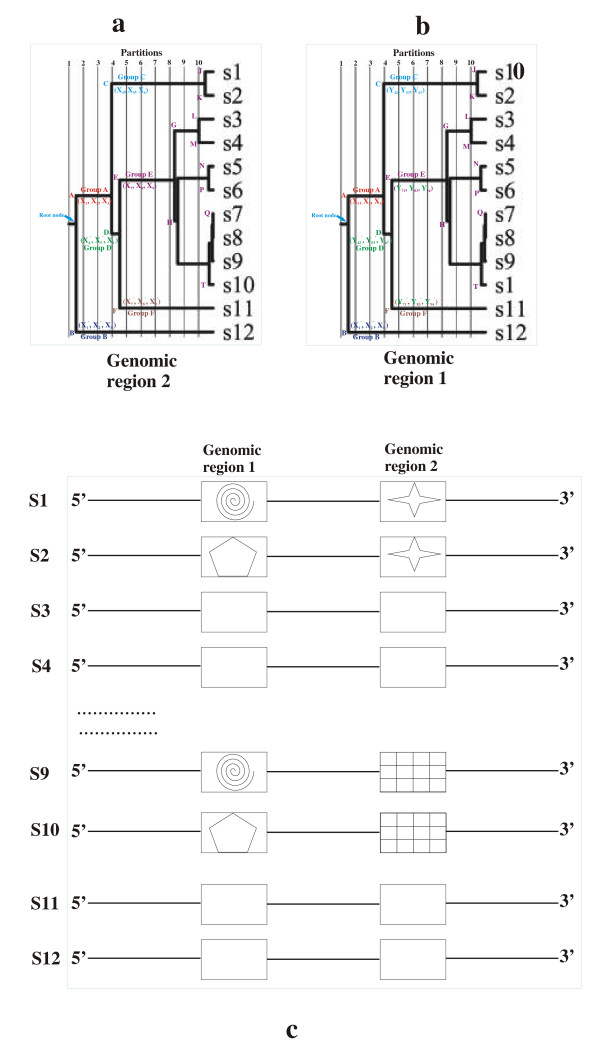
**a, b, c: Illustrating recombination detection using RECOVIR**: (2a) & (2b): Phylogenetic trees corresponding to genomic regions 1 and 2 of sequences s1–s12. The different nodes (A-T), partitions (1–10) and the hypothetical node-distinguishing residues (X_1_, X_2_, ...., Y_1_, Y_2_, ... etc.), are shown. Sequences s1 and s10 have been interchanged to create incongruous trees that depict a simple hypothetical recombination event. (2c): Schematic representation of genomic regions 1 and 2 of sequences s1–s10. Rectangular boxes denote the two regions. Different symbols are used to compare the two genomic regions in sequences s1, s2, s9 and s10 that may cause incongruity between the two trees of figs. (a) and (b). Region 1 is similar in sequence pairs (s1, s9) and (s2, s10) while region 2 is similar in sequence pairs (s1, s2) and (s9, s10). Interchange of region 1 between sequences s2 and s9 would remove the incongruity between the two trees as similar symbols will be brought together in a similar way in both the trees. Blank boxes indicate that those sequences do not cause incongruities between the trees.

Partition-wise comparisons allow RECOVIR to detect abrupt changes in phylogenetic sequence groupings among trees constructed using sequences from different genomic parts. Assuming that absence of recombination creates similar phylogenetic relationships among sequences from different regions of multiple sequence alignments, these abrupt changes or incongruities indicate nodes that may possibly contain recombination sites. For example, an abrupt change is schematically shown using a pair of hypothetical phylogenetic trees constructed using sequences from two different genomic regions of a given set of virus strains (Figs. 2a &2b). A simple incongruity has artificially been built in by interchanging sequences s1 and s10 between the two trees. Consequently, sequences belonging to nodes A and B that are distinguished by residues (X_1_, X_2_, X_3_) and (X_11_', X_21_', X_34_') in the two trees will not define the recombination sites. However, subsequent nodes C, D, E and F which reflect the incongruity between the two trees up to partition 5, will likely define the recombination sites (Figs. 2a &2b). For example, if nodes C and D are distinguished by residues (X_4_, X_5_, X_6_) and (X_4_', X_5_', X_6_') in one part of the genome (Fig. 2a) and by (Y_42_, Y_53_, Y_67_) and (Y_42_', Y_53_', Y_67_') in the other part (Fig. 2b), recombination events must involve some of these residues to explain the incongruity between the trees. Similarly, nodes E and F that are distinguished by residues (X_7_, X_8_, X_9_) and (X_7_', X_8_', X_9_') in one part of the genome (Fig. 2a) and by (Y_71_, Y_83_, Y_94_) and (Y_71_', Y_83_', Y_94_') in the other part must include the recombination sites (Fig. 2b). Similar systematic comparisons of the strain distinguishing residues in nodes G through T of the remaining partitions show that recombination events can not involve sequences s3–s8 and s11–s12 as the topology of the nodes involving these residues are identical in both trees (Figs. 2a &2b). Instead, interchange of region 1 between sequences s2 and s9 may be a possible recombination mechanism that leads to the incongruence between the two trees (Fig. 2c).

In contrast, if there are changes in some of the strain distinguishing residues of a given region without changes in the tree topology, it indicates possible spontaneous mutations. Detection of possible recombination or spontaneous mutations is done manually at present as an automated version of this feature has not yet been built into the software.

#### Program testing and validation

**RECOVIR **was initially validated by identifying the closest strains for five noroviruses and five enteroviruses of known genera from their respective complete and partial amino acid sequences of the capsids (Table 2). These viruses were chosen because of their sequence diversity and the availability of sequences of a large number of strains or serotypes in public databases. The enteroviruses included the complete VP1 sequences of Poliovirus-2 (serotype), human enterovirus (HEV)-B including the Coxsackie viruses, serotypes of the HEV-D species and the simian enteroviruses.

**Table 2:** Strains detected from amino acid sequences of noro and enterovirus capsids

**Input amino acid**				**Output**			
**Complete capsid sequence**		**Partial capsid sequence**		**Detected strains corresponding to input cols**			

**Col (1) Noro**	**Col (2) Entero**	**Col (3) Noro**	**Col (4) Entero**	**Col (1) Noro**	**Col (2) Entero**	**Col (3) Noro**	**Col (4) Entero**

Seacroft [AJ277620]	Polio_2 [DQ841140]	1UK1 [DQ665819]	Cox-B5 [AF114383]	Minireovirus [U02030]	Polio_3 [AF448782]	Beeskow [Q915C5]	Cox-B5 [AF114383]
Appalachicola [AF414406]	Simian_A [NP_714932]	2JP1 [AB264170]	Echo-11 [AB239122]	Chiba [AB042808]	Simian-7 [AF326759]	Chiba [AB042808]	Echo-11 [AF081326]
Baltimore_a [AF414408]	HEV-D [NP_740741]	3JP2 [AB264158]	Echo-13 [AB239091]	Minireovirus [U02030]	HEV-D [D17595]	Norwalk [1IHM]	Echo-13 [AF081327]
Baltimore_b [AF414404]	HEV-71 [AAY59418]	4JP3 [AB264152]	HEV-B [DQ842180]	Chiba [AB042808]	HEV-A71 [AF135944]	Potsdam [AF439267]	Cox-B3 [AF231763]
Boxer [AF538679]	Cox-B5 [AAW71476]	5TP1 [DQ263739]	HEV-75 [DQ468142]	Potsdam [AF439267]	Cox_B5 [AF114383]	Bitburg [AF427112]	Proposed HEV-75 [AF152298]

Input complete capsid sequences are shown in cols 1 and 2 while the input partial sequences are shown in cols 3 and 4. The corresponding closest database strains detected by the program are shown in the last 4 cols whose headers are color matched with those of the input sequence cols. The NCBI accession codes are enclosed within square brackets for all capsid sequences except for Norwalk virus whose PDB code is correspondingly shown. The enterovirus capsid sequences refer to those of VP1 subunits. Abbreviations used: HEV: Human enterovirus; Cox; Coxsackievirus.

RECOVIR was further validated using more than 200 complete and partial sequences of different caliciviruses and more than 100 such picornavirus sequences. Among the calicivirus sequences, nearly 120 were of norovirus strains with the remaining ones being of other caliciviruses. Of the picornavirus sequences, nearly 50 were enterovirus partial sequences including those of echoviruses and other HEV-B serotypes [26], with the remainder chosen from other picornavirus genera and species (Table 1). Most of the partial sequences were randomly chosen from different regions of the capsid with some of the partial sequence lengths being only ~20% of the complete capsid sequence lengths. All norovirus and enterovirus sequences were selected from the NCBI databases [48]. None of the selected sequences were among those from the program databases to minimize possible errors due to biased sequence choices.

### Software description

All functional modules of RECOVIR were written in Perl programming language on Windows XP and the Linux platforms. A Java-based graphical user interface (GUI) has been designed to access all functionalities through user-friendly I/O options (Fig. 1b).

#### Program input and output

Input selections on the GUI have been divided into three categories: sequence, databases and options to control output (Fig. 1b). Any number of query sequences may be input by either pasting them on the white board area or by browsing one or more directories for single or multiple sequence files. There is no limit to the number of input sequence files. The present version reads sequences only in FASTA format ignoring all white spaces and non-alphabet characters.

A dropdown menu in the GUI database section allows specifying the input sequence genus, if known (Fig 1b). A default reference sequence, used for aligning the input sequences and for assigning the aligned location numbers to the input sequence, then pops up in the "Select a reference" box (Fig. 1b). The default reference sequence may be changed if required. In case the genus of the input sequence is not known, the "unknown" option in this box (Fig. 1b) allows the program to automatically determine the genus and reference sequence from the built-in databases.

The "Run" button activates the program, displays a progress bar of the percentage of input sequences processed and displays results in the output section of the GUI (Fig. 1b). Depending on the "View results" options chosen, the output includes only a summary or the complete details of partition-wise matches between the database residues and the residues of the input sequences. Different GUI options allow many other details of such matches to be viewed (Fig. 1b).

## Results and discussion

### Strain identification of complete and partial norovirus capsid sequences

#### Databases

Detailed strain identification results are described here for selected norovirus strains among the caliciviruses and some enterovirus strains among the picornaviruses. Complete details for the other caliciviruses and the picornaviruses can be found with the authors.

**Figure 3:**
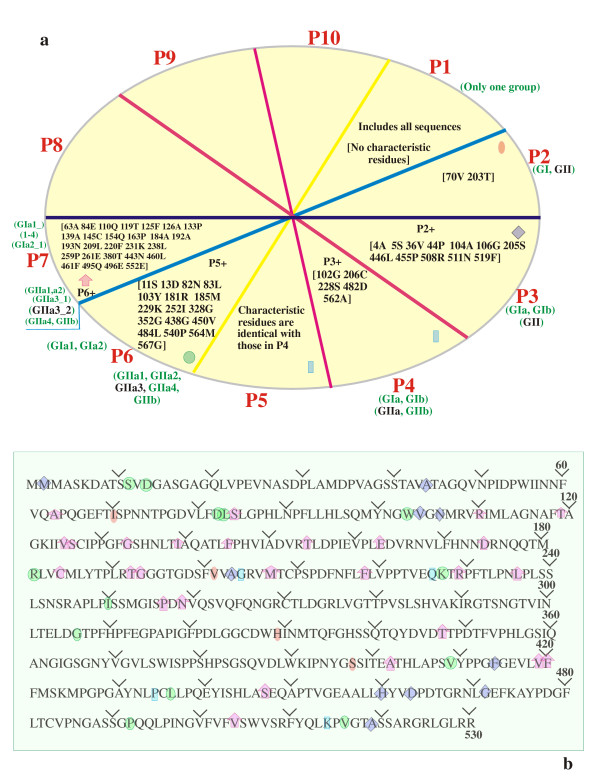
**a, b: Partition-wise matching of norovirus characteristic residues**: (3a): Wheel representation of the 10 partitions P1–P10 showing partition-wise matches for the aligned input target sequence (norovirus "Seacroft"; NCBI accession no. AJ277620). Single letter codes of input target sequence residues that match the characteristic residues in the norovirus database are shown within square brackets for partitions P1–P7. For each partition P1–P6, all sequence groups Gn, where n represents letter combinations, are color coded within parentheses outside the wheel. Of these, sequence groups that match the database characteristic residues are shown in black. Details about the remaining partitions are available with the authors. Symbols Pn+ indicates that all characteristic residues of the previous partition "n" are also included in the current partition. (3b): Detailed partition-wise mapping characteristic residue locations of aligned target sequence (norovirus "Seacroft"; NCBI accession no. AJ277620) on to reference Norwalk virus (PDB ID: 1IHM) sequence. The last residue in each line is numbered and every tenth residue is marked using the  symbol. The symbols , , ,  and  map all of such residues from partitions 1, 2, 3, 4, 5 and 6 respectively of Fig. 2a where these symbols are shown near the edge of each partition.

Sequences belonging to the norovirus genus tree have been described elsewhere [46]. Briefly, these sequences form a single group in partition P1 (Fig. 3a). Partition P2 splits this sequence group into the 2 known major genogroups GI and GII of noroviruses (Additional file 1) [18,46]. Group GI further divides into GIa and GIb in partition P3 while group GII divides into GIIa and GIIb in partition P4 (or P5 that is identical with P4) (Fig. 3a & Additional file 1). Partition 6 further splits both GI and GII groups. Group GIa is divided into GIa1 and GIa2 while group GIIa splits into 4 groups (GIIa1–a4). In partition 7, Group GIa splits into 4 groups GIa1–a4 while GIIa3 splits further into the GIIa3_1 and GIIa3_2 groups (Fig. 3a & Additional file 1). Groups GIb, GIIa4 and GIIb do not split up any further in this partition. Details of the remaining partitions P8–P10 are available with the authors.

#### Characteristic residue comparisons and strain identification for complete sequences

The characteristic residues of the norovirus genus tree stored in the program databases unambiguously identified the closest strains of target norovirus complete capsid sequences (Table 2). For example, comparisons of the characteristic residue locations with the corresponding residues of the norovirus "Seacroft" sequence (NCBI: AJ277620) showed that the maximum number of matches in partition P2 occurred in the aligned locations 70V and 203T of group GII and not for GI (Fig. 3a). The corresponding GI locations were 70I and 201V respectively when mapped to the unaligned reference Norwalk virus (PDB ID: 1IHM) sequence (Fig. 3b). Thus, the program searched only along the GII branch of the norovirus genus tree in subsequent partitions. In partition P3, the query sequence maximally matched the GII characteristic residues 4A, 5S, 36V, 44P, 104A, 106G, 205S, 446I, 455P, 508R, 511N and 519F while the corresponding GI residues in the reference Norwalk virus sequence are 4A, 5S, 36V, 44A, 104V, 106N, 203A, 405L, 414F, 460H, 463D and 471G respectively (Figs. 2a &2b). In partitions P4 and P5, the maximum number of matches (102G, 206C, 228S, 482D, 562A) occurred in GIIa, indicating that further database searches in subsequent partitions should be restricted only to those groups that originate from GIIa.

**Figure 4:**
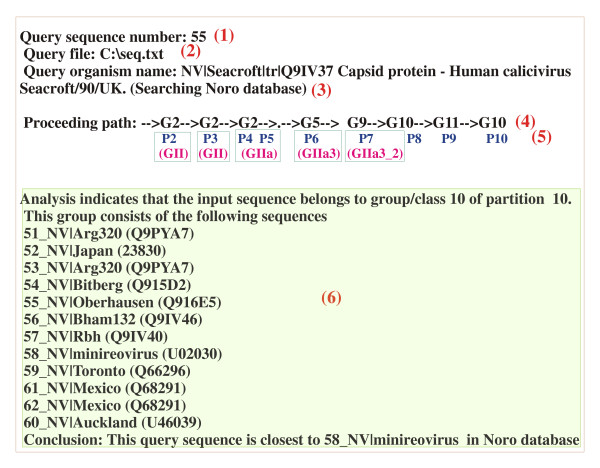
**Sample output from 'overall classification' button in GUI**: Output summary of partition-wise matches of characteristic residues for a typical norovirus query sequence with output line numbers shown within parentheses in red. Details of the query sequence such as its number, input file, organism and sequence accession number are shown in lines 1–3. The partition-wise search results are shown in line 4. Each arrow indicates search progression to partition 'Pn' shown in blue, where n is the partition number. Line 5 shows the matched sequence groups of Fig. 2a within parentheses and in magenta color. The '.' symbol indicates a partition which is identical with the previous one. The block of lines indicated as "6" shows the identified strain of the target sequence and details the sequences of the strain containing group.

Of the 4 possible GIIa choices (GIIa1–a4) in the following partition P6, the maximum number of characteristic residue matches occurred in group GIIa3 (Fig. 3a) corresponding to residues 11S, 13D, 82D, 83L, 103W, 181R, 185M, 227K, 250I, 306G, 329H, 397S, 409V, 436L, 492P, 516V and 519A of the unaligned reference sequence (Fig. 3b). Comparisons in partition P7 showed that of the 2 groups GIIa3_1 and GIIa3_2 originating from the GIIa3 group of the previous partition P6, the GIIa3_2 group maximally matched the characteristic residues (Fig. 3a). These matches occurred at 63A, 84S, 110R, 119T, 125V, 126S, 133G, 139I, 145F, 154T, 163E, 184C, 192T, 193G, 207M, 218F, 229R, 236L, 257P, 259N, 347T, 402A, 419V, 420F, 447S, 448E and 504V of the unaligned reference sequence (Fig. 3b). Similar comparisons in partitions P8–P10 conclusively showed that the query sequence is most similar to the minireovirus-like norovirus strain (Fig. 4)

Similarly, the input Appalachicola Bay, the Baltimore 'a' and 'b' and the Boxer strain sequences most closely resembled the Chiba, minireovirus and the Potsdam strains of noroviruses (Table 2). Strains were also correctly predicted after removing random stretches of 10–15 amino acids from these sequences indicating thereby that the strain prediction capability of the program is quite robust and is independent of the locations of the input target sequences.

#### Strain identification of input partial amino acid target sequences of noroviruses

Strains of five partial amino acid capsid sequences of noroviruses were consistently predicted using both the "noro" and "unknown" database options of the GUI (Fig. 1b). Only one of the 5 input sequences (1UK1) explicitly included the N-terminus residues while the remaining sequences 2JP1, 3JP2, 4JP3 and 5TP1 were from different capsid regions (Table 3).

**Table 3:** Input partial sequences of norovirus capsids and corresponding database matches

**Input target partial capsid sequences of norovirus capsids -A-**						
**>1UK1| **[NCBI:DQ665819]						
MKM**AS**SDANP**S**DGSTANLVPEVNNEVMALEP**V**VGAAIAA**P**V**A**GQQNVIDPWIRNNFVQ**A**P						
**G**GEFT**V**SPRNAPGEILW**SAP**LGPD**L**NPYLSHLAR						
**>2JP1**| [NCBI:AB264170]						
**S**A**DG**ATGAG**Q**LVPEV**N**TADPIPIDP**V**AGSSTAL**A**TAGQVNLIDPWIINNFVQAPQGEFT**I**						
SPNNTPGDV						
**>3JP2| **[NCBI:AB264158]						
GASGAG**Q**LVPEV**N**ASDPLAMDP**V**AGSSTAV**A**TAGQVNPIDPWIINNFVQAPQGEFT**I**SPNNTPGDV						
**>4JP3**| [NCBI:AB264152]						
GTSGAG**Q**LVPEA**N**TAEPISMDP**V**AGAATAV**A**TAGQINMIDPWIMSNFVQAPQGEFT**V**SPNNTPGDV						
>**5TP1| **[NCBI:DQ263739]						
**DG**AAGLVPEINNEAMALDP**V**AGAAIAA**P**L**T**GQQNIIDPWIMNNFVQ**A**P**G**GEFT**V**SPRNSP						
GEVLL**NLE**LGPE**I**						
>**6ST1**| [NCBI: AB058547]						
MMM**AS**KDAPTNMDGTSGAG**Q**LVPEA**N**TAEPISMDP**V**AGAATAV**A**TAGQINMIDPWIMSNF						
VQAPQGEFT**V**SPNNTPGDVLFDLQLGPQLNPFLAHASQ						
>**6ST5**|[Simulated sequence by mutating sequence 6ST1 in locations 11, 30 & 40 highlighted in green)						
MMMASKDAPTSMDGTSGAGQLVPEANTAEQISMDPVAGASTAVATAGQINMIDPWIMSNF						
VQAPQGEFTVSPNNTPGDVLFDLQLGPQLNPFLAHASQ						
						
**-B-**						
**Partial sequence**	**Partition-wise maximally matching characteristic residues (Corresponding sequence group)**					

	**P2**	**P3**	**P4**	**P5**	**P6**	**P7**

1UK1	**70V (GII)**	**4A, 5S**, **36V**, **44P (GII)**			**	**11S**, **46A**, **63A**, **65G**, **82S**, **83A**, **84P**, **89L (GIIa1)**
2JP1	**20Q**, **70I (GI)**	**26N**, **36V**, **44A (GIa)**	--	--	**11S**, **13D**, **14G (GIa1)**	**
3JP2	**20Q**, **70I (GI)**	**26N**, **36V**, **44A (GIa)**	--	--	**	##
4JP3	**	**26N**, **36V**, **44A (GIa)**	--	--	**	##
5TP1	**70V (GII)**	**36V**, **44P (GII)**			##	**13D**, **14G**, **46T**, **63A**, **65G**, **82N**, **83L**, **84E**, **89I (GIIa3_2)**
6ST1	**20Q**, **70V (##)**	**4A, 5S, 26N, 36V, 44A (GIa)**	--	--	**11N 13D 14G 82D 83L (GIa2)**	**46A 63A 65Q 84Q 89L (GIa2_1)**
6ST5	**20Q**, **70V (##) (Synthetic sequence)**	**4A, 5S, 26N, 36V, 44A (GIa)**	--	--	**11S 13D 14G 82D 83L (GIa1)**	**46A 63A 65Q 84Q 89L (GIa1_2 or GIa1_4)**

Ambiguities detected during search process:

**: Equal number of characteristic residue matches found in all of the groups

Blank entry: No matches found for the characteristic residues

--: Identical to previous partition

##: Ambiguity detected: More than one relevant group had equal no. of highest matches

In Table (3B), the maximally matching sequence groups for each partition are shown within parentheses. Residues and sequence groups in Table (3B) use notations of Figs. 2a & 2b. Residues are color matched in Tables (3A) & (3B). One of the mutated residues in the simulated sequence 6ST5 is highlighted in green.

Despite the short sizes of the input partial sequences and the variations in their capsid locations, the program unambiguously recognized the strains of all of them from their matches with the characteristic location residues. Location 70 in partition P2 determined the major genogroup in all but one sequence. Sequences 1UK1 and 5TP1, both containing 70V, belonged to genogroup GII while 2JP1 and 3JP2 belonged to genogroup GI based on the corresponding 70I residue (Table 3; Figs. 2a &2b). All of the GI residues (2JP1 & 3JP2) contained 20Q in partition P2. However, the major genogroup of sequence 4JP3 appeared to be ambiguous as it showed features of both GI and GII groups in partition P2 (ambiguity shown as ** in Table 3). It contained not only 70V, which is typical of GII sequences, but also the GI-indicator 20Q. In addition, this sequence also presented the additional ambiguity of having an equal number of characteristic residue matches in both GI and GII groups for partition P2 (Table 3). To resolve this ambiguity, the program carried over the partition P2 matches of this sequence (4JP3) to partition P3.

In partition P3, the program examined characteristic residue locations 4, 5, 26, 36 and 44 for all of the sequences (Figs. 2a, 2b). Residue 44 unambiguously confirmed the distinction between the GI and the GII group sequences in this partition. Sequences 2JP1, 3JP2 and 4JP3, by virtue of 44A, were all characterized as GI sequences similar to the reference Norwalk virus sequence (Fig. 3b; Table 3). In contrast, sequences 1UK1 and 5TP1, containing 44P, are genogroup GII sequences. In addition, because 1UK1 has 4A and 5S and sequences 2JP1, 3JP2 and 4JP3 have 26N in partition P3, choices of their genogroups were unambiguously confirmed in this partition (Figs. 2a, 2b; Table 3). Residue 36V, being conserved in all of the sequences in P3, was not of much help in determining the sequence groups in this partition (Table 3).

The program ignored partitions P4 and P5 for the 1UK1 and 5TP1 sequences due to ambiguities in residue matches. The next partition P6 showed another ambiguity of having more than one group with the highest number of matches for both these sequences (Table 3). However, matches in partition P7 clearly indicated that sequence 1UK1 belongs to GIIa1 while 5TP1 belongs to group GIIa3_2 (Table 3; Figs. 2a &2b). Further matches in partitions P8–P10 (Table 3) confirmed that 1UK1 and 5TP1 were most similar to the Beeskow and the Bitburg strains respectively (Tables 2, 3; Additional file 1).

Thus, the identified strain of the partial sequence 1UK1 is consistent with its NCBI classification as a member of the GII.4 cluster. However, 5TP1 appears to belong to the GII.3 sequence cluster according to the present analysis and not to GII.4 as shown in the NCBI database. Similarly, partitions P6–P10 allowed the program to confirm that the remaining sequences 2JP1, 3JP2 and 4JP3 indeed belong to genogroup GI (Tables 2 &3) which is consistent with the NCBI classification of these sequences. In addition, the program determined from residue comparisons that these sequences 2JP1, 3JP2 and 4JP3 are most similar to the norovirus GI Chiba, Norwalk and the Potsdam strains respectively (Tables 2 &3). Such detailed strain information is seldom available for partial sequences in public domain databases.

### Strain detection for complete and partial amino acid sequences of enterovirus and other picornavirus capsids

The program first correctly identified the strains for 5 complete and 5 partial enterovirus capsid sequences. The complete capsid sequences were those of the VP1 subunits of poliovirus, simian enterovirus, echovirus and the Coxsackievirus strains (one sequence per strain) (Table 2). Different reference sequences were tested for use in alignment with the target sequences. The target sequence strains were all correctly identified regardless of the choice of the reference sequences (Tables 2 &3). As an example, strain identification for the poliovirus strain (NCBI: DQ841140) using the porcine enterovirus-8 sequence (PEV-8: NCBI accession number AF406813) as reference is briefly described. The second partition P2 of enteroviruses contains only 2 reference groups 1 and 2 in the program database (Table 4). Of these, group 2 contains only the porcine enteroviruses (PEV) serotype 9 (PEV-9; NCBI: AF363453) indicating that the PEV-9 strains are distinct from the other enterovirus strains all of which (including the PEV-8 strain) belong to group 1. This group is characterized by nine residues 40P, 42L, 44A, 46E, 48G, 72E, 124T, 125Y, 127R, 150Q, 155P, 157G, 169W, 176S, 191P, 199Y, 202F and 203Y (reference sequence PEV-8 numbering) in the database (Table 4). Because all of these residues match the corresponding locations in the target poliovirus sequence, the program assigns group 1 to the target sequence in partition P2 and skips the next partition P3 as it is identical to P2 (Table 4).

**Table 4:** Partition-wise strain determination of a poliovirus-2 (PV-2) sequence

**Partition**	**Total no. of groups (Maximally matching group no.)**	**Matching residues (Numbering based on reference PEV-8^¶ ^sequence)**	**Reference database strains present in maximally matching group**
**P2, P3**	2(1)	40P 42L 44A 46E 48G 72E 124T 125Y 127R 150Q 155P 157G 169W 176S 191P 199Y 202F 203Y	All enteroviruses including various human (Coxsackie, echo and polio) strains and some animal strains like bovine and simian enteroviruses. Includes the PEV-8 strain but does not include the PEV-9 strain (More than 100 strains)
**P4**	3(2)	**P3+ **[61L 62Q 78L 106S 115S 131D 152L 170N 174S]	All of the P2/P3 partition strains except PEV-8 strains
**P5**	7(6)	**P4+ **[43T 55G 64T 84L 156H]	32 echoviruses, 21 coxsackieviruses, all polioviruses and 7 other human and animal enteroviruses (Total of ~60 strains)
**P6**	8(7)	**P5+ **[158G]	All polioviruses, 12 coxsackieviruses and 1 echovirus
**P7, P8**	14, 44^£^	**	
**P9**	72(62)	**P6+ **[4K 19L 35N 47T 49E 51D 52T 60E 63A 66C 68F 69S 70L 73T 77Y 79M 80S 81R 83S 85M 90L 109T 113S 117I 119K 120F 123F 126W 129D 137L 138E 140K 153F 154T 166S 167Q 171A 172P 173N 175T 178Y 180R 184C 185P 187S 189R 192F 195V 197N 198Y 206D 207G 209F 216Y 217G 218I 221G 222D 225G 228S 230R 233N 242G 249F 250L 252P 253V 254N 256E 258Y 262P 264V 266Y 268A]	Poliovirus-2 strains
**P10**	75(64)	**P9+ **[11I 17N 33M 36Q 37G 45A 50S 57S 58T 71R 76E 103Y 121K 122A 130L 146N 147L 159A 179T 186A 200T 210D 227I 231M 232A 248I 251R 263L 265S 281P]	Poliovirus-2 strains

**: Ambiguity: 2 or more groups have identical number of matches in both these partitions.

^¶^: Porcine enterovirus (NCBI accession no.: AF406813)

^£^: No matching reference group for partitions P07, P08 due to ambiguity; Strains not shown for these two partitions. All strains of poliovirus serotypes (1, 2 & 3) cluster as an independent group in partition P8.

The complete capsid sequence of input PV-2 target sequence (NCBI accession: DQ841140) was used as input. Symbol **Pn+: **Indicates "including all matching residues of the earlier partition Pn".

The program detects the separation of group 1 into two groups 1a and 1b in partition P4. This creates a total of 3 groups (1a, 1b and 2) in this partition (Table 4). While group 1a contains only the PEV-8 sequences which have independently diverged from group 1 of partition P2/P3, group 1b contains the remaining group 1 sequences of these partitions. Group 2 of partition P2/P3 remains unaltered in partition P4. On comparing the characteristic residues of these three groups, the program determines that the target sequence most closely resemble group 2 in partition P4 because of the matches with residues 61L, 62Q, 78L, 106S, 115S, 131D, 152L, 170N and 174S (PEV-8 numbering) that characterize this group (Table 4). Similarly, matches with residues 43T 55G 64T 84L 156H in partition P5 allow the program to assign group 6 to the target sequence in this partition which contains nearly 60 other similar enterovirus sequences including those of polioviruses (Table 4). Similarly, residue 158G distinguishes group 7 as the maximally matching group out of the eight groups in partition P6. This group includes many similar strains in the program database including 12 strains of different coxsackievirus serotypes, one echovirus strain and strains of all 3 poliovirus serotypes (Table 4).

The three poliovirus serotypes, which remain clustered with several other human enteroviruses in partition P7, finally separate as an independent group in partition P8 (group details not shown). However, partitions P7 and P8 showed an ambiguity with reference to the input target sequence. Both of these partitions have more than one group having an identical highest number of residue matches with the target. This ambiguity did not allow the program to decide the best group in these partitions. Consequently, all group information of these partitions was carried over to the subsequent partition P9 (Table 4).

Partition P9 contains a total of 72 sequence groups including an independent group (#62) containing only the poliovirus-2 strains. The program detected this group to be the maximally matching group for the target sequence and this detection was confirmed in the next partition P10. Thus, the input target sequence was correctly detected as a poliovirus-2 strain (Table 4). Strains for the remaining picornaviruses (Tables 2 &3) were also unambiguously detected regardless of the choice of the reference strains.

### Spontaneous mutations and recombination

#### Spontaneous mutations

Partition-wise matches for the norovirus partial sequence 6ST1 indicated spontaneous mutations. While residue 20Q in partition P2 indicates that 6ST1 belongs to GI, residue 70V in the same partition indicates that this sequence may belong to GII as seen for sequence 5TP1 (Table 3). The program, therefore, checks both these possibilities for maximum number of matches in subsequent partitions. Each subsequent partition confirms that this sequence indeed belongs to GI and is closest to the Potsdam strain (NCBI accession AF439267). Therefore, location 70, which is an isoleucine for other GI sequences (Table 3), may be a spontaneous mutation site given the relatively large number of matches found which, in turn, are expected to maintain the topology of the tree. However, a complete sequence would be required to completely rule out the involvement of this site in recombination events.

Residue-wise comparisons may suggest that a few spontaneous mutations may be sufficient to force RECOVIR to detect a wrong genogroup of the target sequences. For example, it may appear that the single mutation 44A to 44P in sequence 6ST1 might switch it to a GII group (Table 3). However, the fact that the program checks the maximum number of matches of strain diversifying residues in each partition ensures that such single mutations within the capsid does not randomly change the strain type of the sequence. This is one of the major strengths of the present method.

For the software to branch off to a different strain on the phylogenetic tree, several mutations are usually required. For example, if locations 11, 30 and 40 of sequence 6ST1 are mutated without changing the rest of the sequence, it results in the synthetic GI sequence 6ST5 belonging to group GIa1 in partition P6 instead of GIa2 that corresponded to 6ST1 (Table 3; Additional file 1). Comparisons of strain diversifying residues in further partitions result in sequence 6ST5 belonging to a different GI cluster than that of 6ST1 (data not shown). Thus, the present method is able to identify spontaneous mutations that may lead to subtle changes in the phylogenetic groups of partial sequences.

#### Recombinations

Only a few enterovirus strains could be analyzed for recombination using RECOVIR due to the manual nature of such analysis in the present version of the software. Partition-wise comparisons of strain diversifying residues of VP1–VP3 genes of the hepatitis-A (HAV) strains at the nucleotide and the amino acid levels indicated that the SLF88 (AY032861) and the MBB (M20273) strains may be putative parental strains that yield the recombinant product strain 9F94 (AJ519487) (data not shown). This is consistent with recent results obtained using conventional topology comparisons of phylogenetic trees by sliding windows across the corresponding multiple aligned sequences [55].

Despite the limited results, it appears that the present approach may be more efficient in detecting recombination sites than current methods which are inherently time consuming as their accuracy depends on detailed topological comparisons obtained from multiple runs using different sequence windows. In addition, the entire procedure must be repeated every time a new sequence needs to be analyzed. In contrast, the present method does not repeatedly compare phylogenetic trees using sliding sequence windows. Instead, it efficiently performs node-wise comparisons of the trees using a one-time created database of strain distinguishing residues.

### Approximate processing times

The program rapidly identified the calicivirus and the picornavirus strains. In typical runs on outbreak sequence data available from public databases, it took only 6 and 9 minutes of real time respectively to identify the strains of a number of norovirus sequences contained in 2 files. The first file had 71 complete norovirus capsid sequences while the second file had 117 norovirus capsid sequences of which 22 were complete sequences while the remaining 95 were partial sequences each of which was between 75 and 85 residues long. This indicates a real time (including I/O) of approximately 5 seconds per sequence. Comparable speeds were also observed in processing mixtures of calicivirus and picornavirus sequences when the "unknown" database option (Fig. 1b) was required. Complete benchmark details of such strain identification runs are available with the authors.

### Advantages of the software

#### Trees based on complete capsid sequences are robust

A significant computational advantage of using complete capsid sequences to construct the RECOVIR databases is that these databases are very robust with respect to the method used to construct the database trees. The overall topology of the trees, described by the relative branch lengths and the connectivity among the branches, remain essentially unchanged regardless of the neighbor-joining method used to draw the tree. For example, in all of the trees constructed using the complete capsid sequences, the sequence groups and the characteristic residues of the groups remain unchanged for partitions P2–P9. Only the P10 sequence groups show minor changes depending on the tree construction method used (data not shown but are available with the authors). Consequently, strain identification results using RECOVIR are very reliable.

In contrast, trees constructed using a partial capsid sequences or using amino acids of more conserved and relatively smaller non-capsid regions (such as the polymerase or the non-coding parts of the virus genomes) are relatively unstable because the topology of these trees critically depend upon the method of tree construction. Therefore, databases generated from such trees are not as reliable as the ones generated using trees of complete capsid sequences.

#### Tree-structured databases

The partitioned residue databases in RECOVIR confer important advantages to this software over existing homology-based strain identification techniques. Because each partition and its nodes highlight branching orders that provide evolutionary context of sequence relationships, the databases in RECOVIR most likely indicate biologically significant residues as seen for the noroviruses [46].

More practically, searches using the database residues are able to show increasing similarities between the target sequences and appropriate database sequences as subsequent partitions are considered. Consequently, ambiguities in strain identification, such as those occurring due to the partial nature of the sequences or those due to possible mutations in the target sequences, may be resolved at least in the first few partitions where appropriately matched sequence groups are found. Such matches are not possible using conventional homology based sequence comparisons.

#### Advantages in classification

Residues of the RECOVIR databases may be used to determine relatedness among the corresponding sequence groups in a partition-dependent manner thus helping in virus classification. For example, while the database residues of P2 distinguish among two of the major norovirus groups and the murine norovirus group, the corresponding residues in subsequent partitions distinguish among the more closely related sequence groups. Such residue-based distinctions among the sequence groups provide useful ways to uniquely identify many tentatively classified sequences like those of the bovine and the alphatron noroviruses.

#### Bovine and the alphatron norovirus groups

**Figure 5:**
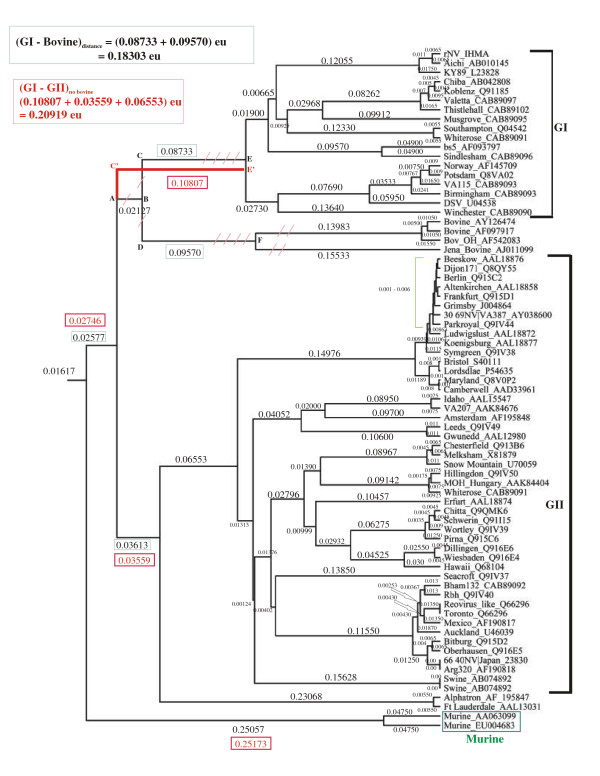
**Phylogenetic tree of noroviruses with and without the bovine sequences**: The names and NCBI accession number are shown for each strain. The GI, GII and the murine sequence groups are indicated. The evolutionary units (eu) are the distances in terms of number of mutations. The tree that includes the bovine norovirus sequences is shown in black while changes in tree topology due to the exclusion of the bovine sequences are shown in red. Red slashes indicate the bovine branches AB, CD, CE and DF that get excluded in the tree constructed without the bovine sequences. Instead, branch C'E' connects the GI group to the GII group. Distances which undergo the maximum changes are enclosed within green boxes while the corresponding changed distances are enclosed within red boxes. All other distances remain nearly identical in both trees. The bigger green and the red boxes at the top left hand corner of the figure show calculations of the GI-bovine distance in one tree and the GI-GII distance in the other tree. The green square bracket indicates a cluster of GII sequences whose branch lengths are within the values shown. The sequences used in the trees are identical with those used in earlier analysis (Chakravarty et al., 2005). Both trees were drawn using the same neighbor-joining methods under identical distance models and sequence alignment parameters as described in the text.

Our earlier analysis had indicated that the bovine noroviruses may belong to genogroup I while the alphatron-like sequences may belong to genogroup II [46]. One way to understand this is to compare the inter-group evolutionary distances, measured in evolutionary units (eu), between GI, GII and the groups containing the bovine and the alphatron sequences. These distances refer to the number of mutations among the sequences. In trees constructed using neighbor-joining methods and Gonnet-250 distance matrices, the GI-GII distance, calculated between the GI and the GII groups after excluding the bovine sequences, is 0.20919eu while the corresponding GI-bovine distance, calculated between the GI and the bovine groups, is 0.18303eu (Fig. 5). Thus, the difference between these distances (0.02586eu) is less than the partition width which results in clustering the bovine sequences together with the GI sequences in the same P2 group. Similarly, the GI-GII distance, calculated after excluding the alphatron-like sequences, is more than the GII-alphatron distance calculated between the GI and the alphatron groups thereby clustering the alphatron-like sequences in the GII group of partition P2. Thus, the phylogenetic distances are consistent with the proposal that the bovine sequences appear to share sequence similarities with the GI genogroup while the alphatron sequences share GII sequence features [46].

**Figure 6:**
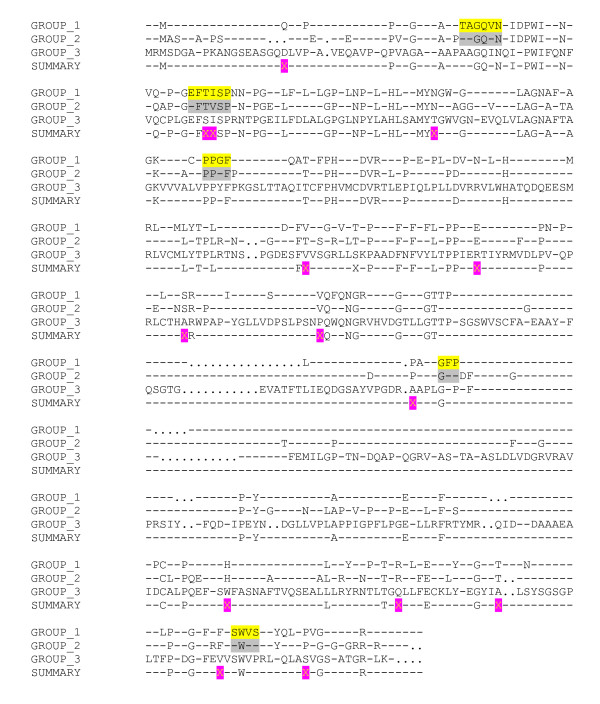
**Alignment of the three norovirus sequence groups of partition P2**: GROUP_1 consists of the aligned GI sequences that include the bovine sequences while GROUP_2 consists of the aligned GII including the alphatron sequences. Group_3 consists of the murine sequences. All amino acid residues are shown using single letter alphabets. Dashes (-) indicate variable locations and dots (.) indicate insertions/deletions. The SUMMARY lines indicate the alignment results of these two groups. The X, highlighted in magenta, denote group-specific locations i.e. locations which are conserved within each group but not among the groups. Such locations distinguish among the different sequence groups and form part of the RECOVIR databases. The yellow highlighted regions denote some residues that are conserved in GI and the bovine sequences but are not identical with the corresponding GII locations. The corresponding locations that are conserved in GII and the alphatron-like sequences are highlighted in grey.

These relative phylogenetic distances among the sequence groups may be further understood by comparing the sequences of the P2 groups. On aligning the three different P2 sequence groups, the conserved residues among the bovine sequences show significant similarities with those of the GI sequences but not with the conserved sequences of GII. For example, the conserved GI residues 45T, 46A, 47G, 48Q, 49V and 50N (Norwalk virus numbering) are also conserved in the bovine sequences (Fig. 6). Similarly, the alphatron sequences show more residue similarities with the GII sequences than with those of GI (Fig. 6). Such similarities are further confirmed in subsequent partitions P3–P10 of RECOVIR. Therefore, the software places the bovine-like sequences in GI and the alphatron-like sequences in the GII group. Although these assignments are consistent with our earlier analysis [46], they do not agree with recent sequence clustering studies that assign independent genogroups to the bovine and the alphatron sequences [40]. Experimental determination of the possible biological roles of the conserved residues (Fig. 6) will help resolve the issue.

#### Other advantages

Many of the RECOVIR database residues are involved in putative capsid related functions in noroviruses [46]. This indicates that these residues and the corresponding sequence groups may critically identify subtle changes in capsid related functions among the genogroups, species and serotypes. Hence, RECOVIR may provide functional insights not currently available with current classification schemes of caliciviruses and picornaviruses.

#### Software limitations

A limitation of the present version of the software is that the implemented residue databases may become outdated given the rapidly increasing number of emerging strains of calici and picornaviruses. Therefore, the databases need to be regularly upgraded. However, upgrades should be carried out only when changes become statistically significant. In our experience, this would mean that at least 50% of the emerging sequences must show changes in the same residue locations before those residues are considered reliable enough to be incorporated in the databases.

## Conclusion

A software package RECOVIR that can efficiently and accurately characterize strains of the highly divergent caliciviruses and picornaviruses is described. In contrast to current techniques that largely rely on sequence similarity scores to identify strains, RECOVIR implements a method indicated previously for noroviruses [46]. The software creates partitioned databases of capsid residues that unambiguously distinguish among a large number of calicivirus and the picornavirus reference strains. Using efficient tree-based search techniques, residues of target capsid sequences are compared with the residue databases to rapidly identify strains of the target sequences of all caliciviruses and picornaviruses. Strains of more than 300 complete and partial capsid sequences of calici and picornaviruses were successfully identified with an average time (real time including I/O) of approximately 5 seconds per sequence. The method is general enough to be applicable to the nucleotide sequences of calici and picornavirus capsids thereby providing rapid and powerful alternatives that complement current strain determination and classification techniques of these and other ssRNA viruses.

## Availability and Requirements

• **Project home pages: **

• **Operating systems: **Windows-XP and Linux

• **Programming language: **Perl and Java

• **Other requirements: **X-Windows support (such as Cygwin) is needed for remotely running the program under Linux environment.

## Competing interests

The author(s) declares that there are no competing interests.

## Authors' contributions

DZ coded the software and designed the GUI. SC developed the concept, designed the algorithm and its implementation and created the initial databases. SC and DZ performed extensive troubleshooting with both synthetic and real data. SC and GEF wrote the paper. All authors suggested improvements at different stages of manuscript preparation and read and approved the final version of the manuscript.

## Supplementary Material

Additional file 1 Partitioned distribution of norovirus strains. Supplementary Table showing detailed distribution of norovirus strains in different sequence groups for partitions P2–P7. Click here for file

## References

[B1] Drake JW, Holland JJ (1999). Mutation rates among RNA viruses. Proc Natl Acad Sci USA.

[B2] Farkas T, Deng X, Ruiz-Palacios G, Morrow A, Jiang X (2006). Development of an enzyme immunoassay for detection of sapovirus-specific antibodies and its application in a study of seroprevalence in children. J Clin Microbiol.

[B3] Coiras MT, Aguilar JC, Garcia ML, Casas I, Perez-Brena P (2004). Simultaneous detection of fourteen respiratory viruses in clinical specimens by two multiplex reverse transcription nested-PCR assays. J Med Virol.

[B4] Corless CE, Guiver M, Borrow R, Edwards-Jones V, Fox AJ, Kaczmarski EB, Mutton KJ (2002). Development and evaluation of a 'real-time' RT-PCR for the detection of enterovirus and parechovirus RNA in CSF and throat swab samples. J Med Virol.

[B5] Grondahl B, Puppe W, Hoppe A, Kuhne I, Weigl JA, Schmitt HJ (1999). Rapid identification of nine microorganisms causing acute respiratory tract infections by single-tube multiplex reverse transcription-PCR: feasibility study. J Clin Microbiol.

[B6] Read SJ, Kurtz JB (1999). Laboratory diagnosis of common viral infections of the central nervous system by using a single multiplex PCR screening assay. J Clin Microbiol.

[B7] Jokela P, Joki-Korpela P, Maaronen M, Glumoff V, Hyypia T (2005). Detection of human picornaviruses by multiplex reverse transcription-PCR and liquid hybridization. J Clin Microbiol.

[B8] Atmar RL, Estes MK (2001). Diagnosis of noncultivatable gastroenteritis viruses, the human caliciviruses. Clin Microbiol Rev.

[B9] Glass RI, Noel J, Ando T, Fankhauser R, Belliot G, Mounts A, Parashar UD, Bresee JS, Monroe SS (2000). The epidemiology of enteric caliciviruses from humans: a reassessment using new diagnostics. J Infect Dis.

[B10] Hansman GS, Guntapong R, Pongsuwanna Y, Natori K, Katayama K, Takeda N (2006). Development of an antigen ELISA to detect sapovirus in clinical stool specimens. Arch Virol.

[B11] Honma S, Nakata S, Kinoshita-Numata K, Kogawa K, Chiba S (2000). Evaluation of nine sets of PCR primers in the RNA dependent RNA polymerase region for detection and differentiation of members of the family Caliciviridae, Norwalk virus and Sapporo virus. Microbiol Immunol.

[B12] Vinje' J, Hamidjaja RA, Sobsey MD (2004). Development and application of a capsid VP1 (region D) based reverse transcription PCR assay for genotyping of genogroup I and II Noroviruses. J Virol Methods.

[B13] Vinje J, Deijl H, van der Heide R, Lewis D, Hedlund KO, Svensson L, Koopmans MP (2000). Molecular detection and epidemiology of Sapporo-like viruses. J Clin Microbiol.

[B14] Green KY, Chanock RM, Kapiakan AZ (2001). Human caliciviruses.

[B15] Stanway G, Brown F, Christian P, Hovi T, Hyypiä T, King AMQ, Knowles NJ, Lemon SM, Minor PD, Pallansch MA, Palmenberg AC, Skern T, Fauquet CM, Mayo MA, Maniloff J, Desselberger U, Ball LA (2005). Family *Picornaviridae*. Virus Taxonomy. Eighth Report of the International Committee on Taxonomy of Viruses. Virus Taxonomy Eighth Report of the International Committee on Taxonomy of Viruses.

[B16] Matson DO, Szucs G (2003). Calicivirus infections in children. Curr Opin Infect Dis.

[B17] Buchen-Osmond C, ed (2003). Caliciviridae.

[B18] Green KY, Chanock RM, Kapiakan AZ (2001). Human caliciviruses. Fields virology.

[B19] Green KY, Vinje J, Gallimore CI, Koopmans M, Hale A, Brown DWG (2000). Capsid protein diversity among Norwalk-like viruses. Virus Genes.

[B20] Ando T, Noel JS, Fankhauser RL (2000). Genetic Classification of "Norwalk-like Viruses. The Journal of Infectious Diseases.

[B21] Katayama K, Shirato-Horikoshi H, Kojima S, Kageyama T, Oka T, Hoshino F, Fukushi S, Shinohara M, Uchida K, Suzuki Y, Gojobori T, Takeda N (2002). Phylogenetic analysis of the complete genome of 18 Norwalk-like viruses. Virology.

[B22] Schuffenecker I, Ando T, Thouvenot D, Lina B, Aymard M (2001). Genetic classification of "Sapporo-like viruses". Arch Virol.

[B23] Okada M, Yamashita Y, Oseto M, Ogawa T, Kaiho I, Shinozaki K (2006). Genetic variability in the sapovirus capsid protein. Virus Genes.

[B24] Robinson S, Clarke IN, Vipond IB, Caul EO, Lambden PR (2002). Epidemiology of human Sapporo-like caliciviruses in the South West of England: molecular characterisation of a genetically distinct isolate. J Med Virol.

[B25] Wirblich C, Meyers G, Ohlinger VF, Capucci L, Eskens U, Haas B, Thiel HJ (1994). European brown hare syndrome virus: relationship to rabbit hemorrhagic disease virus and other caliciviruses. J Virol.

[B26] IAH Virus pages. http://www.iah.bbsrc.ac.uk.

[B27] Hadfield AT, Lee W, Zhao R, Olivera MA, Minor I, Rueckert RR, Rossmann MG (1997). The refined structure of human rhinovirus 16 at 2.15 A resolution: implications for the viral life cycle. Structure.

[B28] Kim SS, Smith TJ, Chapman MS, Rossmann MG, Pevear DC, Dutko FJ, Felock PJ, Diana GD, McKinlay MA (1989). Crystal structure of human rhinovirus serotype 1A (HRV1A). J Mol Biol.

[B29] Rossmann MG, Arnold E, Erickson JW, Frankenberger EA, Griffith JP, Hecht HJ, Johnson JE, Kamer G, Luo M, al AGMe (1985). Structure of a human common cold virus and functional relationship to other picornaviruses. Nature.

[B30] Verdaguer N, Blaas D, Fita I (2000). Structure of human rhinovirus serotype 2 (HRV2). J Mol Biol.

[B31] Zhao R, Pevear DC, Kremer MJ, Giranda VL, Kofron JA, Kuhn RJ, Rossmann MG (1996). Human rhinovirus 3 at 3.0 A resolution. Structure.

[B32] Sherry B, Mosser AG, Colonno RJ, Rueckert RR (1986). Use of monoclonal antibodies to identify four neutralization immunogens on a common cold picornavirus, human rhinovirus 14. J Virol.

[B33] Sherry B, Rueckert RR (1985). Evidence for at least two dominant neutralization antigens on human rhinovirus 14. J Virol.

[B34] Oberste MS, Maher K, Kilpatrick DR, Pallansch MA (1999). Molecular Evolution of the Human Enteroviruses: Correlation of Serotype with VP1 Sequence and Application to Picornavirus Classification. J Virol.

[B35] Prasad BVV, Hardy ME, Dokland T, Bella J, Rossmann MG, Estes MK (1999). X-ray Crystallographic Structure of the Norwalk Virus Capsid. Science.

[B36] Chen R, Neill JD, Estes MK, Prasad BVV (2006). X-ray structure of a native calicivirus: Structural insights into antigenic diversity and host specificity. PNAS.

[B37] Jiang X, Wang M, Graham DY, Estes MK (1992). Expression, self-assembly, and antigenicity of the Norwalk virus capsid protein. J Virol.

[B38] Kobayashi S, Sakae K, Suzuki Y, Ishiko H, Kamada K, Suzuki K, Natori K, Miyamura T, Takeda N (2000). Expression of recombinant capsid proteins of chitta virus, a genogroup II Norwalk virus, and development of an ELISA to detect the viral antigen. Microbiol Immunol.

[B39] Kobayashi S, Sakae K, Suzuki Y, Shinozaki K, Okada M, Ishiko H, Kamata K, Suzuki K, Natori K, Miyamura T, Takeda N (2000). Molecular Cloning, Expression, and Antigenicity of Seto Virus Belonging to Genogroup I Norwalk-Like Viruses. J Clin Microbiol.

[B40] Zheng D-P, Ando T, Fankhauser RL, Beard RS, Glass RI, Monroe SS (2006). Norovirus classification and proposed strain nomenclature. Virology.

[B41] Kageyama T, Kojima S, Shinohara M, Uchida K, Fukushi S, Hoshino F, Takeda N, Katayama K (2003). Broadly reactive and highly sensitive assay for for Norwalk-like viruses based on real-time quantitative reverse transcription PCR. J Clin Microbiol.

[B42] Richards GP, Watson MA, Kingsley DH (2004). A SYBR green, real-time RT-PCR method to detect and quantitate Norwalk virus in stools. J Virol Methods.

[B43] Fankhauser RL, Monroe SS, Noel JS, Humphrey CD, Bresee JS, Parashar UD, Ando T, Glass RI (2002). Epidemiologic and Molecular Trends of "Norwalk-like Viruses" Associated with Outbreaks of Gastroenteritis in the United States. J Infect Dis.

[B44] Kageyama T, Shinohara M, Uchida K, Fukushi S, Hoshino FB, Kojima S, Takai R, Oka T, Takeda N, Katayama K (2004). Coexistence of multiple genotypes, including newly identified genotypes, in outbreaks of gastroenteritis due to norovirus in Japan. J Clin Microbiol.

[B45] Rozanov M, Plikat U, Chappey C, Kochergin A, Tatusova T (2004). A web-based genotyping resource for viral sequences. Nucleic Acids Res.

[B46] Chakravarty S, Hutson AM, Estes MK, Prasad BVV (2005). Evolutionary Trace Residues in Noroviruses: Importance in Receptor Binding, Antigenicity, Virion Assembly, and Strain Diversity. J Virol.

[B47] Lichtarge O, Bourne HR, Cohen FE (1996). An evolutionary trace method defines binding surfaces common to protein families. J Mol Biol.

[B48] Life sciences databases at the National Center for Biotechnology Information (NCBI), USA. http://www.ncbi.nlm.nih.gov/.

[B49] Thompson JD, Higgins DG, Gibson TJ (1994). CLUSTAL W: improving the sensitivity of progressive multiple sequence alignment through sequence weighting, position-specific gap penalties and weight matrix choice. Nucl Acids Res.

[B50] Gonnet GA, Cohen MA, Benner SA (1992). Exhaustive matching of the entire protein sequence database. Science.

[B51] Felsenstein J (2004). Inferring Phylogenies.

[B52] Innis CA, Shi J, Blundell TL (2000). Evolutionary trace analysis of TGF-beta and related growth factors: implications for site-directed mutagenesis. Protein Eng.

[B53] Saitou N, Nei M (1987). The neighbor-joining method: a new method for reconstructing phylogenetic trees. Mol Biol Evol.

[B54] Tamura K, Dudley J, Nei M, Kumar S (2007). MEGA4: Molecular Evolutionary Genetics Analysis (MEGA) Software Version 4.0. Mol Biol Evol.

[B55] Costa-Mattioli M, Ferre V, Casane D, Perez-Bercoff R, Coste-Burel M, Imbert-Marcille BM, Andre EC, Bressollette-Bodin C, Billaudel S, Cristina J (2003). Evidence of recombination in natural populations of hepatitis A virus. Virology.

