# Supplementary material for: RECOVIR: An application package to automatically identify some single stranded RNA viruses using capsid protein residues that uniquely distinguish among these viruses
Source: BMC Bioinformatics. 2007 Oct 10;8:379. doi: 10.1186/1471-2105-8-379 (PMC2174958; doi:10.1186/1471-2105-8-379)
Supplement: Additional file 1 — Partitioned distribution of norovirus strains. Supplementary Table showing detailed distribution of norovirus strains in different sequence groups for partitions P2–P7. [file 1471-2105-8-379-S1.pdf]

Partitions	Sequence groups	Norovirus sequences in groups
P2	GI	Norwalk_1IHM, Aichi_Aic[AB010145), Kyoto89_Ky89[L23828), Chiba_Chb[AB042808), Koblenz_Kob[Q91I85), Valetta_Val[CAB89102), Thistlehall_Thi[CAB89102), Musgrove_Mus [CAB89095), Southampton_Sou[Q04542), Whiterose_Wh1[CAB89091), bs5_bs5[AF093797), Sindlesham_Si [CAB89096), Norway Stav_Nor[AF145709),Potsdam_Pot[Q8VA02), VA115_Va1[AY038598), Birmingham_Bi[CAB89093), DSV_Sa[U04538), Winchester_Wi[CAB89090), Jena_Bovine_Jen[AJ011099), Bovine_BO [AF542083)
	GII	Beeskow_Bee [Q915C5), Dijon171_Dij[Q8QY55), Berlin_Ber[Q915C2), Altenkirchen_Al[Q916E8), Frankfurt_Fr[Q915D1), Grimsby_Gr [AJ004864), VA387_Va3[AY038600), Parkroyal_Pa[Q9IV44), Ludwigs_Lu[Q915C9), Koenigs_Ko[Q915C4), Symgreen_Sy[Q9IV38), Bristol_Br[S40111), Lordsdale_Lo[P54635), MD145_Md[Q8V0P2), Camberwell_Ca[Q68537), Idaho_Id[Q913B7), VA207_Va2[Q91H09), Amsterdam_Am[AF195848), Leeds_Le[Q9IV49), Gwynedd_GW[AAL12980), Chesterfield_Ch[Q913B6), Mexico[Q68291), Snow Mountain_Sn[U70059), Hillingdon_Hi[Q9IV50), MOH_MO[AF397156), White River_Wh2[AF414423), Erfurt_Er[Q915C7), Chitta_Cht[Q9QMK6), Schwerin_Sc[Q91I15), Wortley_Wo[Q9IV39), Pirna_Pi[Q915C6), Dillingen_Di[Q916E6), Wiesbaden_Wi[Q916E4), Hawaii_Ha[Q68104), Seacroft_Se[Q9IV37), Bham132_Bh[Q9IV46), Rbh_Rb[Q9IV40), Minireo_Re[U02030), Toronto_To[Q66296), Melksham_Mel[X81879), Auckland_Au[U46039), Bitburg_Bi[Q915D2), Ober_Ob[Q916E5), Japan_Ja[23830), Arg320_Ar_Mex[Q9PYA7), Swine_Sw1[Q8V713), Swine_Sw2[BAB83516), Alphatron_Al[AF195847), Ft Lauderdale_Ft[AAL13031)
P3	GIa	1IHM, Aic, Ky89, Chb, Kob, Val, Thi, Mus, Sou, Wh1, bs5, Si, Nor, Pot, VA1, Bi, Sa, Wi
	GIb	Jen, BO
	GII	Bee, Dij, Ber, Al, Fr, Gr, Va3, Pa, Lu, Ko, Sy, Br, Lo, Md, Ca, Id, Va2, Am, Le, GW, Ch, Mex, Sn, Hi, MO, Wh2, Er, Cht, Sc, Wo, Pi, Di, Wi, Ha, Se, Bh, Rb, Re, To, Mel, Au, Bi, Ob, Ja, Ar, Sw1, Sw2, Al, Ft
P4 & P5	GIa & Ib	Same as in P3
	GIIa	Bee, Dij, Ber, Al, Fr, Gr, Va3, Pa, Lu, Ko, Sy, Br, Lo, Md, Ca, Id, Va2, Am, Le, GW, Ch, Mex, Sn, Hi, MO, Wh2, Er, Cht, Sc, Wo, Pi, Di, Wi, Ha, Se, Bh, Rb, Re, To, Mel, Au, Bi, Ob, Ja, Ar, Sw1, Sw2
	GIIb	Al, Ft
P6	GIa1	1IHM, Aic, Ky89, Chb, Kob, Val, Thi, Mus, Sou, Wh1, bs5, Si
	GIa2	Nor, Pot, VA1, Bi, Sa, Wi
	GIIa1	Bee, Dij, Ber, Al, Fr, Gr, Va3, Pa, Lu, Ko, Sy, Br, Lo, Md, Ca
	GIIa2	Id, Va2, Am, Le, GW
	GIIa3	Ch, Mex, Sn, Hi, MO, Wh2, Er, Cht, Sc, Wo, Pi, Di, Wi, Ha, Se, Bh, Rb, Re, To, Mel, Au, Bi, Ob, Ja, Ar
	GIIa4	Sw1, Sw2
	GIIb	Al, Ft
P7	G ((Ia1_1)) to [Ia1_4]	((1IHM, Aic, Ky89)) (Chb, Kob, Val, Thi, Mus) { Sou, Wh1} [bs5, Si]
	GIIa1 & a2	Same as in P6
	GIIa3_1	Ch, Mex, Sn, Hi, MO, Wh2, Er, Cht, Sc, Wo, Pi, Di, Wi, Ha
	GIIa3_2	Bh, Rb, **Re**, To, Mel, Au, Bi, Ob, Ja, Ar
	GIIa4 & IIb	Same as in P6

Supplementary Table 1: Norovirus strains in different sequence groups in partitions P2-P7. This distribution is based on norovirus capsid residues. Partition P2 entries show complete strain names along with their NCBI accession numbers enclosed within square brackets and strain codes that are indicated as "_X" where X refers to the 2 or 3 letter codes e.g X id 1IHM for Norwalk strain and it is Aic for the Aichi strain. These strain codes are used in all subsequent partitions P3-P7. The different GIa1 sequence groups in P7 are enclosed in different types of parentheses: GIa1_1: ((..)); GIa1_2: (..); GIa1_3: {..}; GIa1_4: […]. The red boldfaced code in P7 indicates the minireovirus strain (NCBI accession no. U02030).
